# Greece since the 1960s: the mortality transition revisited: a joinpoint regression analysis

**DOI:** 10.1007/s12546-023-09301-2

**Published:** 2023-02-22

**Authors:** Konstantinos N. Zafeiris

**Affiliations:** Laboratory of Physical Anthropology, Department of History and Ethnology, Democritus University of Thrace, P. Tsaldari 1, 69132 Komotini, Greece

**Keywords:** Greece, Mortality transition, Life expectancy, Probabilities of death, Cluster analysis, Modal age at death, Mode, Left and right inflexion points, Gini coefficient, Joinpoint analysis, Non-linear regression

## Abstract

Mortality transition in Greece is a well-studied phenomenon in several of its aspects. It is characterised by an almost constant increase in life expectancy at birth and other ages and a parallel decrease in death probabilities. The scope of this paper is a comprehensive assessment of the mortality transition in Greece since 1961, in the light of holistic analysis. Within this paper, life tables by gender were calculated and the temporal trends of life expectancy at several ages were examined. Moreover, a cluster analysis was used in order to verify the temporal changes in the mortality patterns. The probabilities of death in large age classes are presented. Furthermore, the death distribution was analysed in relation to various parameters: the modal age at death, mode, left and right inflexion points and the length of the old age heap. Before that, a non-linear regression method, originating from the stochastic analysis, was applied. Additionally, the Gini coefficient, average inter-individual differences, and interquartile range of survival curves were examined. Finally, the standardised rates of the major causes of death are presented. All the analysis variables were scholastically examined for their temporal trends with the method of Joinpoint Regression analysis. Mortality transition in Greece after the year 1961 is asymmetrical with a gender and an age-specific component, leading to the elevation of life expectancy at birth over time. During this period, the older ages’ mortality decreases, but at a slower pace than that of the younger ones. The modal age at death, mode, the left and right inflexion points and the width of the old age heap denote the compression of mortality in the country. The old age death heap shifts towards older ages, while at the same time, the variability of ages at death decreases, being verified by the Gini Coefficient and average inter-individual differences. As a result, the rectangularization of survival curves is evident. These changes have a different pace of transition over time, especially after the emergence of the economic crisis. Finally, the major causes of death were the diseases of the circulatory system, neoplasms, diseases of the respiratory system and others. The temporal trends of these diseases differ according to the diseases and gender. Greece’s mortality transition is an asymmetrical stepwise process characterised by its gender and age-specific characteristics. This process, despite being a continuous one, is not linear. Instead, a combination of serious developments over time governs the country’s modern mortality regime. The evaluation of Greece’s mortality transition through the lens of more advanced analytical methods may provide new insights and methodological alternatives for assessing mortality transition in other countries of the world.

## Introduction

Since Valaoras’s research (1936, 1960), mortality and its transition in Greece have been thoroughly investigated. Several subsequent analyses demonstrated the almost constant increase in average longevity in several ages (see Siampos, 1994, 2003; Kotzamanis, 2000). These developments follow the decrease in infant and child mortality, temporal changes in the ages of the accident hump and developments in middle-aged and older adults’ mortality (see: Zafeiris & Kostaki, 2019; Zafeiris et al., 2017; Kotzamanis et al., under publication; for maternal mortality see: Dimitrakakis et al., 2001). As a result, mortality and health transitions have moved ahead in Greece compared to the other countries of south-eastern Europe (Zafeiris & Skiadas, 2017). On the contrary, the mortality regime prevailing in the country has more similarities with western and southern Europe (Kotzamanis & Zafeiris, 2021; Zafeiris, 2019).

In Greece, there are regional variations in mortality patterns and causes of death (see Kalogirou et al., 2012). In the twenty-first century, Northern Greece and Attica presented the lowest average longevity rates, whereas the islands of the Aegean and Crete presented the highest (Zafeiris et al., 2018; see also: Tsimbos et al., 2011, 2014). In addition, there is significant differentiation between Greece’s local population and immigrants, which is related to differences in mortality conditions observed within their countries of origin and their low socio-economic status (Verropoulou & Tsimbos, 2016).

According to Nikolaidis et al. (2004), between 1967 and 1996, the reduction of communicable diseases led to an increase in average longevity followed by a gradual increase in the prevalence of degenerative diseases. However, according to later research, communicable diseases played a minor role in regulating longevity since1994 onwards. In contrast, the four most important causes of death were the diseases of the circulatory system (in decreasing order), neoplasms (almost unchanged over time), diseases of the respiratory system and external causes of morbidity and mortality (Kotzamanis et al., under publication; see also: Zafeiris, 2020a, 2020b). In similar studies, the decreasing trend of avoidable mortality is also emphasised between 1980 and 2007 (Ollandezos et al., 2011).

From 2008 onwards, Greece faced a severe financial and socio-economic crisis that dictated an investigation of the crisis’ effects on mortality. Some scholars indicated a “crude mortality” increase in 2009–2015 that did not only derive from the economic crisis but also from population ageing (Vardakas et al., 2019). According to this approach, the citizens of southern Greece, the elderly, and women suffered the most. Another essential characteristic was the increase in suicides during this era (Alexopoulos et al., 2019). Other researchers link 1/3 of the observed additional deaths in 2012 with austerity (Vlachadis et al., 2014), while infant and child mortality rates did not differ among the pre-crisis and crisis period, except for a transient increase in 2009–2010 (Michas et al., 2014). In a recent study, Siahanidou et al. (2019) showed that the once prevailing trend of decreasing infant mortality stopped approximately four years after the onset of the economic crisis. It should be added that Greece’s health system has been significantly affected by austerity policies, which caused the deceleration of amenable mortality decrease and *“increased mortality from several conditions amenable to medical interventions”* (Zilidis et al., 2020).

Meanwhile, Zafeiris and Kostaki (2019) showed that the economic crisis and the general improvement of infrastructure positively affected the accident hump (corresponding to ages 15–30 or more years), mainly in the male individuals of the country. Kotzamanis and Zafeiris (2021; see also: Kotzamanis et al., under publication) observed a recent deceleration of improvements in the average longevity in Greece due to the minor survival improvements in almost all ages after the emergence of the economic crisis, except for infant mortality, which is a particular case (see later in the text). In general, various indices suggest a “cause and effect” relationship between deep recession and mortality at certain ages and/or in connection with specific causes. However, the authors recognise that in order to assess the relevant effects on mortality (except for extreme cases, i.e., the total collapse of the socio-economic system of former socialist countries), it is necessary to have more extended time series available.

In conclusion, despite the fact that the mortality transition is a well-studied phenomenon, there is a general lack of a comprehensive and modern description of the phenomenon (except for a small number of publications, such as Zaferiris, 2019; Kotzamanis & Zafeiris, 2021), which consider the latest mortality developments since the moment that relatively reliable data are available. Additionally, the temporal trends of the mortality transition have not been meticulously investigated. On the contrary, a general description is provided that lacks their precise estimation. Therefore, this paper aims to revise the mortality transition in modern Greece, a twofold effort. Firstly, it focuses on analysing mortality trends in Greece from 1961 onwards by using up-to-date, sophisticated methods (i.e., the Gini coefficient, left and right inflexion points of the death density distribution and others; see Data and Methods section). Secondly, by applying the Joinpoint Regression Analysis, it aims to assess and evaluate the actual temporal trends of mortality transition in Greece.

The Joinpoint Regression Analysis (Kim et al., 2000, 2004) examines whether the trends of a given quantity vary in different segments of time, and a statistical model is created to summarise them (see, for example, Chatenoud et al., 2015; Rea et al., 2017; Missikpode et al., 2015). Time (expressed as years, months, days and other quantities) is the independent variable, while the dependent variable is the one whose temporal trends are analysed. This analysis creates a series of successive linear regression lines (called segments), each connecting with the next one at a point of time (joinpoint), which denotes the change of the temporal trends of the dependent variable. These lines have a different annual percentage change of the dependent variable (and slope), and thus the mortality transition is described in detail (for more details, see Data and Methods along with the Results sections of this paper).

It should be noted that since 2020 the coronavirus pandemic afflicted the country, leading to a significant deterioration in mortality trends without following a previously existing pattern. Thus, the present analysis stopped in 2019. A future publication will present the trends in the COVID-19 era. The other reason is the lack of official data for 2020–2021 during this research. Therefore, this paper focuses on the period between 1961 and 2019.

## Data and methods

The employed data was derived from the Eurostat database (https://ec.europa.eu/eu-rostat/data/database): population of January 1^st^ of each year by gender and age, the relevant deaths and major causes of death. Based on these data, the following analytical methodology will be applied in order to analyse the trends between 1961 and 2019, the two extreme years for which data are available in the Eurostat database:

### Calculation of full life tables

The full life tables of males and females were constructed after Chiang (1979), and life expectancy at birth and the ages of 15, 30, 45 and 65 years was examined.

### The joinpoint regression analysis

The Joinpoint Regression Program (2021) was used to analyse the temporal trends of these variables (dependent variables) to examine any differences in the pace or the timetable of transition and to evaluate their characteristics. Time is the independent variable, expressed in years. This software, created by the National Cancer Institute of the United States of America (Division of Cancer Control and Population Sciences; see https://cancercontrol.cancer.gov/about-dccps), uses trend data and fits the simplest joinpoint model that data allow.

Initially, a minimum number of joinpoints is assumed (in this analysis, 0 joinpoints), and the other ones are added when they are statistically significant. This process creates a sequence of linear equations (segments) over time. The significance tests use a Monte Carlo Permutation Method. In order to perform the analysis this software considers that models are linear on the log of each variable. However, this is not connected with the visualisation of the results in figures, in which the relevant axis can be either in ordinary (like the life expectancy at birth), or in a logarithmic scale (like the probabilities of death). An *Annual Percentage Rate of Change (APC)* is calculated for each segment and its slope. However, the latter is not cited in this paper as it describes the changes identical to that of *APC*. Afterwards, an overall rate of change for all segments, named *Average Annual Percent Change* (*AAPC*), is estimated. For more details of this method, see the software webpage (https://surveillance.cancer.gov/join-point/) and Kim et al., (2000, 2001). This method will be applied to all variables used in this paper and described in the following paragraphs.

### Decomposition of the year to year changes of life expectancy at birth

After estimating the different segments of change of life expectancy at birth with the joinpoint analysis, an Arriaga (1984, 1989) decomposition procedure will be applied for each of them. Based on the full life tables, each year will be compared within these segments with the next one to estimate how the age-specific mortality differences contribute to the overall changes in average longevity. The results, represented as within each segment averages, will be cited for the following age groups: 0, 1–14, 15–29, 30–44, 45–64, 65+, i.e., for infancy, childhood and early adolescence, late adolescence and early maturity (corresponding to the accident hump). The people from the age of 30 and over are separated into two groups: the working population and seniors (i.e., mainly pensioners).

### The probabilities of death

The probabilities of death will be analysed for the large age classes of the previous paragraph. The utilisation of one-year length age classes is inadequate due to the random fluctuations found in the first and exploring phases of the analysis. Instead, the analysis of larger age groups “smoothed” these fluctuations and allowed a better and more precise depiction of the temporal trends of mortality patterns.

Additionally, people aged more than 65 years will be studied in two larger age groups. The first one will be the 65–84 years group and the other one the 85–99 years. It has to be noted that the open-ended age interval is the 85+ years in the life tables of the period 1961–1985. Afterwards, it is the 100+ years. Thus, it was decided to analyse the temporal trends in the age group of 65–84 years, for which data were available for the whole analysis period (1961–2019). Secondly, people aged 85+ in 1986 and the subsequent years were born at the beginning of the twentieth century, when the civil registration system was absent in Greece. Additionally, many of them were born in places outside the contemporary borders of the country (Greece at the beginning of the twentieth century was in size less than a half of its current area). Thus, any data for these age groups are far more problematic than the younger ages. In that way any findings for the age group 85–99 years that will be cited in this paper must be treated with caution. Several other data quality problems will be discussed in the Results section of this paper.

Afterwards, following Zafeiris and Tsoni (2021), a cluster analysis was conducted based on these death probabilities to identify any segmentation of the mortality patterns throughout the transition process. During this procedure, while examining the correlation matrix of the variables, a multicollinearity test revealed that the variables (i.e., the probabilities of death) were highly correlated (the Variance Inflation Factor was well above 10 for both genders). Consequently, this might distort the underlying construction of the dendrograms of cluster analysis. In order to solve this problem, the distance measure used was the Mahalanobis one, as it is not affected by multicollinearity. The required clustering procedure for the constructions of dendrograms was the unweighted pair-groups averages, which had the best cophenetic correlation coefficient (for the relevant terminology, see Zafeiris & Tsoni, 2021).

Finally, temporal trends and levels for each of the probabilities of death were examined by applying the joinpoint analysis procedure.

### The life table’s death and survival curves

1. The left and right inflexion points of the death distribution

**Fig. 1:**
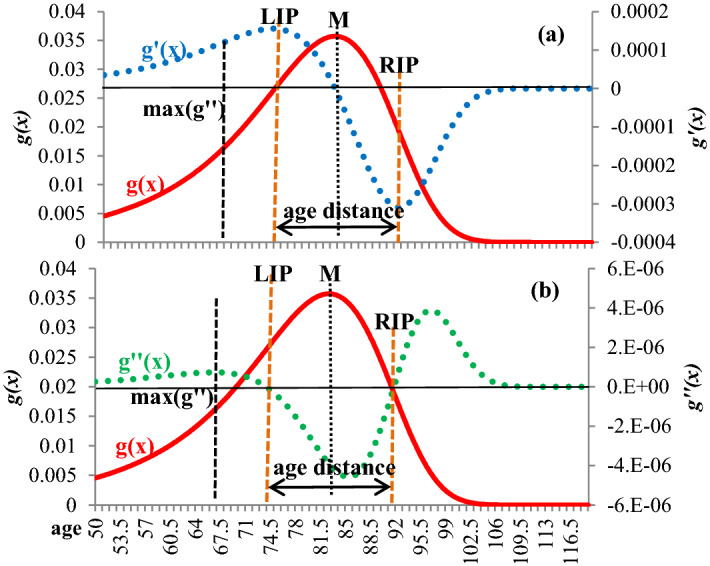
Left and right inflexion points.* Source*: Zafeiris and Skiadas (2017)

In order to calculate several indices of the mortality curve, a method of non-linear regression originating from stochastic analysis (first exit time theory) was applied. According to this method, the death density distribution is initially calculated and then normalised for being comparable between different populations. Afterwards, the following death density function *g(x)* can be fitted to the data (for the proof and the relevant calculations see Skiadas & Skiadas, 2010, 2013, 2014; Jansen & Skiadas, 1995; see also: http://www.cmsim.net/id13.html):

1 $$g\left(x\right)=k (l+\left(c-1\right)(bx{)}^{c})({{x}^{-3/2}} {e}^{-\frac{(l-(bx{)}^{c}{)}^{2}}{2x}})$$

where *x* is the age and *c, b, l, k* parameters to be estimated. The relevant coefficient of determination of the fit R^2^ was consistently higher than 0.9 in both genders. After smoothing this curve, three essential characteristics of the mortality curve in the old age heap will be estimated:

- *The modal age at death M* corresponds to the age at which the age-at-death distribution reaches its maximum, that is, the age where most of the deaths are occurring (Canudas-Romo, 2008, 2010). *M* represents an alternative longevity indicator in the modern era because infant mortality is very low, and the deaths are concentrated in the old age heap. Unlike life expectancy at birth, the modal age at death is not affected by infant, young, middle and old adult mortality (see, for example, Horiuchi et al., 2013; Canudas-Romo, 2010).
- The *old-age death heap’s height or mode* corresponds to the *g(x)* value at the modal age at death.
- *The width of the old age death heap* (age distance in Fig. 1): This width can be estimated in several ways, like the Kannisto indicators (2000), but in this paper, a dynamic procedure is used based on the inflexion points of the death curve. In mathematical terms, an inflexion point corresponds to a smooth curve point where the curvature changes sign. In order to find it, it is necessary to calculate the first and second derivatives of a function, in this case of the *g(x)* function described by Eq. (1). In simple terminology, the first derivative tells us whether a function increases or decreases and to what degree. The second derivative gives the same information for the first derivative. The first derivative of Eq. (1) will then show a rate of change of *g(x)* between successive ages, known here as *“speed of the death distribution”*. The second derivative will be the rate of change of the speed of death distribution, and it will be called *“acceleration”*. The *g(x)* curve has a left and a right inflexion point in the old age heap, found in this paper with one decimal precision as happened with the other two characteristics (for the calculations see http://www.cmsim.net/id13.html).

The left inflexion point (*LIP*) corresponds to the age of maximum speed of death distribution (*g*′*(x)*; Fig. 1) when health is rapidly burdened (Fig. 1a). After the LIP, the speed decreases and tends to be 0 at the modal age of death. Before the *LIP*, the acceleration (*g*′′*(x)*) attains its first maximum. The *g*′′*(x)* is positive before the LIP, but afterwards, it declines to 0 in the *LIP*. After the *LIP*, the acceleration becomes negative, and the speed of death distribution decreases, reaching its minimum at the right inflexion point (*RIP*). At the same time, *g(x)* values after modal age at death decrease. After the *RIP* point, acceleration becomes positive (Fig. 1b), and the death speed increases up to some age and then remains stable (Fig. 1a). The age distance between the left and the right inflexion points (*RIP–LIP*) will estimate the width of the mortality curve at the old age heap (for the whole procedure, see Zafeiris & Skiadas, 2017).

In that way, the verticalisation property of the distribution of deaths by age will be examined, corresponding to the concentration of deaths around the relevant modal age (see Cheung et al., 2005).

2. The gini coefficient

The Gini Coefficient (index) is an index of diversity or inequality, directly related to the Lorenz curve, which represents *“the graphical relationship between the cumulative normalised rank of the population from the poorest to the richest and the cumulative normalised wealth held by these population from the poorest to the richest”* (Sitthiyot & Holasut, 2021). Thus, the Gini Coefficient is the area between the diagonal line denoting full equity and the Lorenz curve (see Lorenz, 1905).

In mortality analysis, it expresses the inter-individual variability in the age at death (see, for example, Le Grand, 1987; Wilmoth & Horiuchi, 1999), being the mean of the absolute differences of the individuals’ length of life to the average length of life in a population (Shkolnikov et al., 2003). This coefficient equals zero when all individuals die at the same age. It becomes one if all people die at age 0, and one individual dies at an infinitely old age (Shkolnikov et al., 2003). The higher the Gini Coefficient, the greater the diversity of people’s length of life. In contrast, if this coefficient decreases over time, the age dispersion of deaths decreases too. As a result, deaths increasingly concentrate around the modal age at death, and consequently, the Gini Coefficient is a measure of rectangularization of the survival curves. Additional to the Gini Index, the average inter-individual differences in length of life will be calculated, to examine in absolute values the magnitude of these differences in the male and female population of the country. Calculations were done according to Shkolnikov et al. (2003).

3. The quartiles of the survival curve and the interquartile range

In addition to the approaches described in the previous two subparagraphs, the analysis of the quartiles and the interquartile range of the survival curves, being ubiquitous measures of inequality, will verify the findings of the Gini Coefficient and the age width between the left and right inflexion points (see Shkolnikov et al., 2003 for the calculations).

For estimating the values of measures in subparagraphs 1, 2, and 3, the full life tables from 1986 onwards will be used. The open-ended interval for these life tables is 100+ years, not 85+ as before. In the latter, besides any data problems, most of the estimates were above 85 years, which posed questions for the validity of results. So, they were omitted from the analysis.

### Major causes of death

Finally, the temporal trends of the standardised rates (see: Preston et al., 2001, pp. 21–37) of the major causes of death will be examined in the light of joinpoint analysis. These causes are the diseases of the circulatory system, neoplasms, diseases of the respiratory system, external causes of morbidity and mortality, diseases of the digestive system and certain infectious and parasitic diseases. All of them are the most crucial regulating factors of the mortality regime in Greece. All other diseases will be summed up in the “all others” category.

## Results

### Life expectancy

**Fig. 2:**
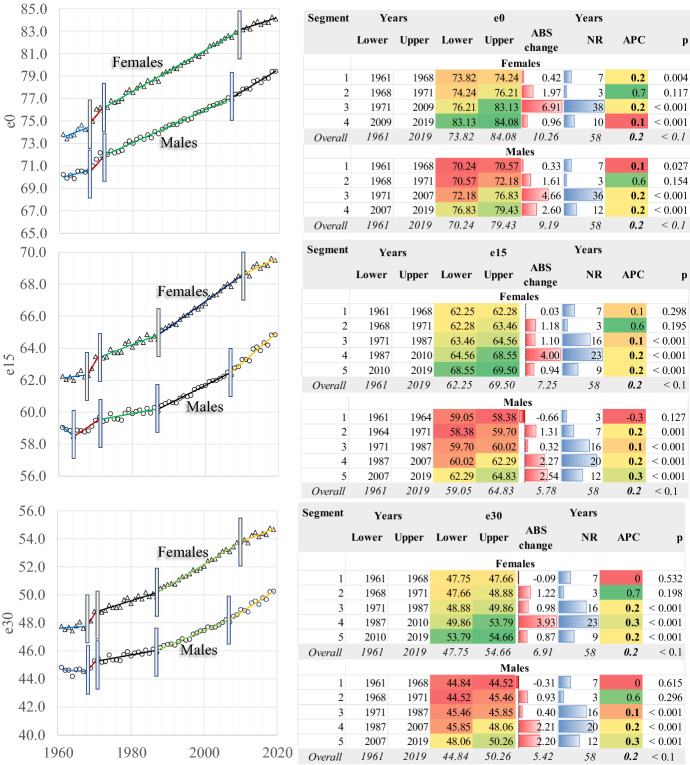
Life expectancy at birth, 15 and 30 years. Greece 1961–2019. AAPC values are stored in row “Overall” and column “APC”

The mortality transition in Greece (Fig. 2) is characterised by an almost continuous improvement of the average longevity (life expectancy at birth, e0) between 1961 and 2019; males gain 9.2 years and females 10.26. Thus, women have a longer life span, and their longevity improvement is more pronounced in absolute numbers. However, the pace of change of the average longevity differs over time, having a gender-specific element besides the identical overall AAPC for both males and females (0.2%).

The Joinpoint Regression Analysis revealed that between 1961 and 1968, any longevity changes were minimal: in absolute numbers less than 0.5 years in men and women, with an Annual Percent Change (APC) of 0.1% in males and 0.2% in females. Between 1968 and 1971, e0 changes by 1.61 years in males and 1.97 in females, but the probability that APC was different from zero is insignificant. The average longevity “jumps” in females in this period while it has a “smoother” improvement in males. However, considering that data quality is questionable before the mid-1980s, such a finding must be treated with caution.

The most significant developments observed within the recent mortality transition in Greece come in the period 1971–2007 in males and 1971–2009 in females. The relevant gains in life expectancy at birth are 4.66 and 6.91 years, respectively (APC 0.2% and 0.25%). The transition slows down after 2009 in women (APC 0.13%), as they have reached significantly high e0. In contrast, it accelerates in males (APC 0.25%), reaching from 76.83 the 79.43 years.

It is worth noting that this period coincides with the vast economic crisis which afflicted Greece after 2008. The deterioration of this trend a few years after the crisis is temporary, and eventually, the regression line of this segment shows that longevity continues to increase. To conclude, based on the data available so far, the economic crisis does not seem to have significantly affected the longevity of the population of the country.

**Fig. 3:**
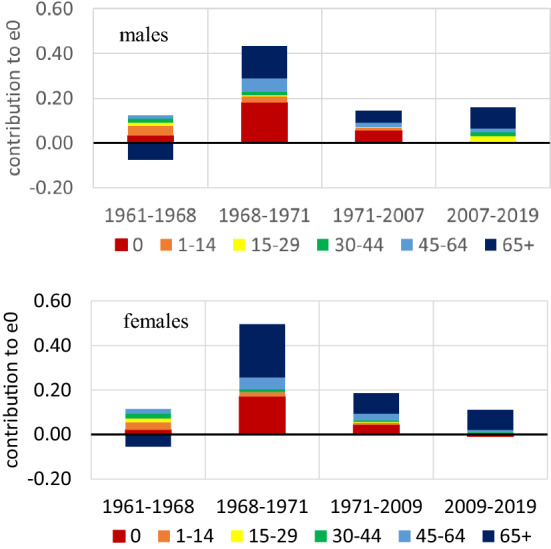
Decomposition of life expectancy at birth (e0) in large age classes. Averages for time periods

The decomposition of life expectancy at birth with Arriaga’s method (1984, 1989) denotes the significant shifts in Greece’s mortality regime (Fig. 3). In 1961–1968, the most critical regulating factor of life expectancy developments was the mortality of the older ages, which negatively affects e0 as it increases over time. In contrast, all the other age groups have an evenly distributed positive effect. In the next period, despite the fact that all ages contribute positively, the mortality of infants and of people aged 45+ years regulates the longevity developments. The same happens in the next period of 1971–2007 or 1971–2009, but the effects are smaller than previously. After 2007 in males and 2009 in females, any longevity developments result from mortality changes in the older (65+) ages. All other ages play a minor role, especially infant mortality, which becomes extremely low.

Therefore, as the mortality transition in Greece progresses, it begins to be determined almost exclusively by the mortality of the older ages because both infant mortality and that in the younger ages become gradually very low. This phenomenon is known in the literature as “rotation of mortality” (Li et al., 2013). In such a situation, mortality transition firstly relates to the decline of infant and childhood mortality and, in some cases, middle-age mortality. Afterwards, these improvements slow down, and the mortality decrease in older people accelerates. In Greece, this phenomenon is considered in some publications to be strong in both genders (see Vékás, 2020) and will be discussed later.

Similar observations in life expectancy at birth can be made for the other ages in Fig. 2. Of them, the significant acceleration of longevity in males aged 15 and 30 years old after 2007 must be noted. The same happens with females aged 15 years while for those aged 30 years the higher increase of life expectancy is found in the 1987–2010 period. This phenomenon will be discussed later in the text.

**Fig. 4:**
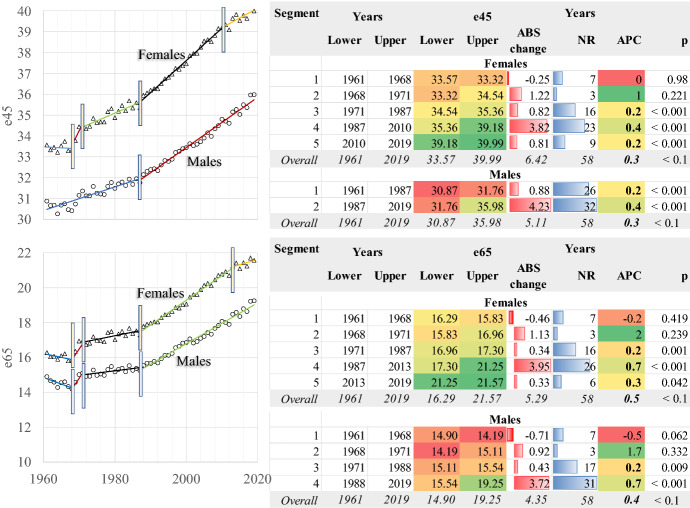
Life expectancy at ages 45 and 65 years. Greece 1961–2019. AAPC values are stored in row “Overall” and column “APC”

Mortality transition is straightforward at 45 years of age (Fig. 4; AAPC = 0.3%). E45 increases in two periods in males: 1961–1987 (APC = 0.2) and 1987–2017, when it accelerates, increasing annually by 0.4%. In females, a more complicated pattern persists in which the highest pace of increase is in the period 1987–2010. Afterwards, as e45 is high, any improvements decelerate. Longevity trends are more complicated for the older ages (65+). In both genders, longevity decreases in the first period (1961–1968) and increases with various rhythms afterwards. The maximum in females was in 1987–2013 (APC = 0.7%), reaching 21.25 years. Afterwards, it decelerates. In males, it has increased constantly since 1988, reaching 19.25 years in 2019.

Comparing these two ages with the previous ones described in Fig. 2, the average life expectancy is increasing faster here. However, as revealed by females, a slowdown in trends is observed when the transition is well advanced. The time point in which this happens has gender and age-specific components, as described previously.

### The probabilities of death

**Fig. 5:**
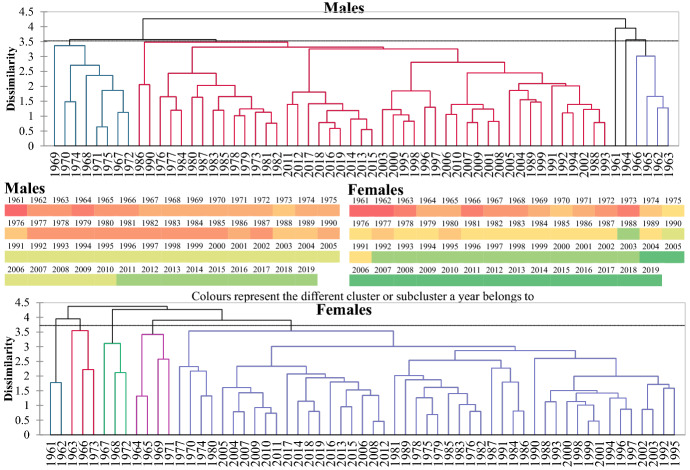
Cluster analysis of the mortality patterns. Cophenetic correlation coefficient: Males = 0.7 Females = 0.8

Cluster Analysis revealed the progressive differentiation of mortality patterns over time in both genders (Fig. 5). The formed clusters branch into subdivisions, namely subclusters, which in their turn, contain groups of higher affinities, and this process continues until the identification of the most similar groups. The heat maps of Fig. 5 represent the major clusters of the dendrogram until 1975 and the first subclusters afterwards.

In both genders, it is evident that the most diversified mortality patterns are before the year 1975. This diversification has already been identified after examining the temporal trends of life expectancy at birth and other ages. The variability in this period is higher in females. After 1975, all years form a large cluster, which embraces smaller subclusters.

The first subcluster of this period consists of the years up to the end of the 1980s. However, the diversity remains significant as many years within this period belong to different subclusters in both genders. The next period in the development of mortality patterns in Greece starts in the early 1990s when the two genders start to deviate significantly. For males, the period 1991–2010 forms a distinct but variable subcluster, with minor dissimilarities between the years and the modern era starting in 2011. In females, in which the mortality transition proceeded faster, this era started in 2004. Meanwhile, a more homogeneous group consists of the years 1992–2003.

**Fig. 6:**
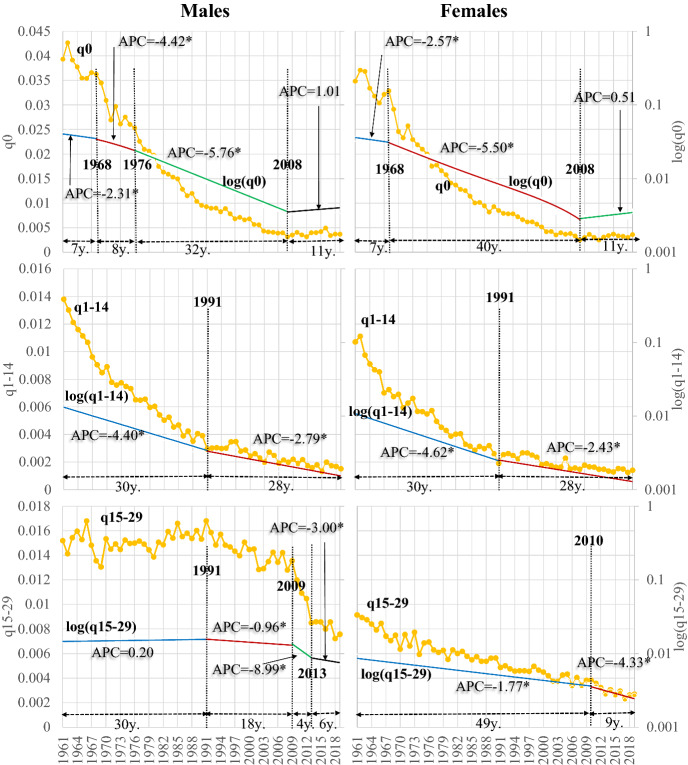
Probabilities of death at ages < 30. *Asterisk denotes statistical significance at 0.05 level

**Fig. 7:**
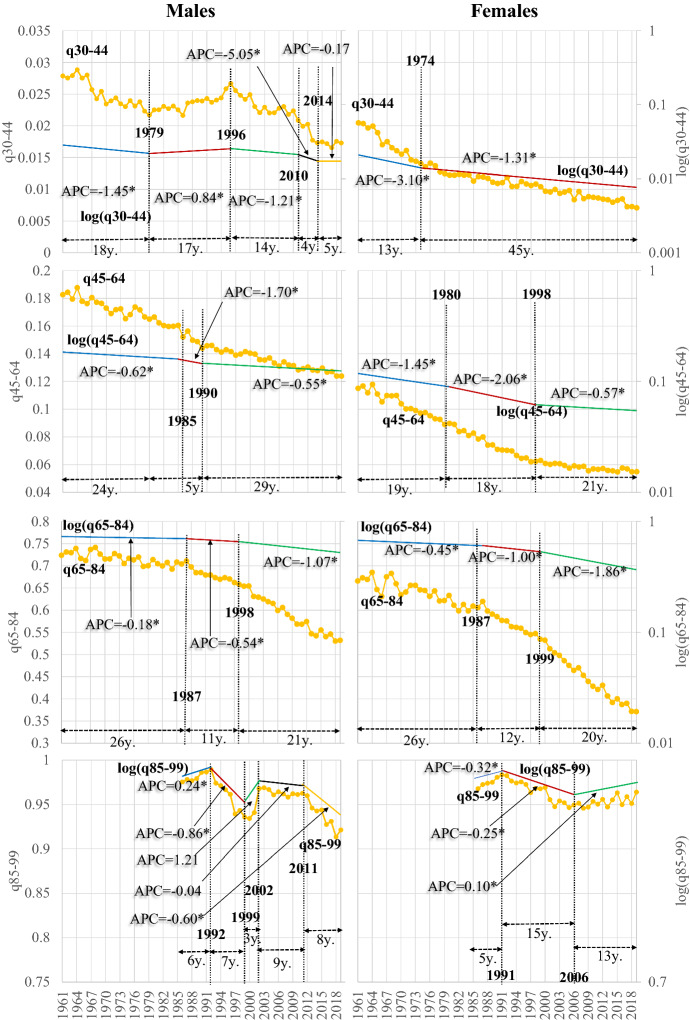
Probabilities of death at ages >  = 30. *Asterisk denotes statistical significance at 0.05 level

Thus, a stepwise transition of mortality patterns is observed in Greece, according to which, besides their affinities, the two genders have significant differences. These differences will be pictured in detail after analysing the temporal trends of the probabilities of death of large age classes (except the first one, which refers to infant mortality) seen in Figs. 6 and 7.

In both genders and all age classes, the probabilities of death tend to decrease over time (Figs. 6 and 7), with different timetables and paces of decrease, in a way that an asymmetric scheme emerges. This asymmetry has an age and a gender-specific component, including the differentiated temporal trends between the older and younger ages and the specialisation of these trends in the two genders. The effects of these changes on average longevity have been discussed in the previous paragraphs.

Infant mortality follows a four-step transition in males (Fig. 6). The joinpoints that define these steps are 1968, 1976 and 2008. During this process, the transition accelerates after 1968, reaching a maximum APC (− 5.75%) between 1976 and 2008. After 2008, while being very low, infant mortality tends to increase (+ 1.01% yearly), a possible effect of the economic crisis on infant survival. The same happens in females, where the transition is slightly different, having a three-step process and slightly lower rates of change. Thus, the first characteristic of mortality transition in Greece is the fast decline of infant mortality over time.

This pattern of fast decrease also holds for the mortality of the children aged 1–14 years, but the transition has a slightly slower pace and comes in two periods in both genders. Before 1991 both genders have a faster transition, which decelerates but is still faster than most older ages (see Fig. 7).

On the contrary, in the ages 15–29 years, corresponding to the accident hump, the transition has different characteristics among men and women. Indeed, the relevant probabilities do not change much until 1991, when they start to decrease by about 1% per year. Considering that most mortality in these ages comes from lifestyle risk factors (traffic accidents, exposure to psychotropic substances and other agents), it is evident that several aspects of everyday life improved over time: one could suggest road infrastructure, better cars and others. However, until 2009 when this period ended, the problem remained very significant for males. Afterwards, the economic crisis limited their economic potential, with risky behaviours consequently being limited. At the same time, infrastructure was becoming even better. The annual changes between 2009 and 2013 were about − 10%, constituting the fastest pace of transition for both genders and all ages. This trend continued later but at lower rates of change. In females, the decline is faster than males before 2009–2010, but at the same time, these rates were lower. Finally, the exact reasons applying for males caused the rapid decline of their mortality after 2010 (APC = −4.3%).

The transition is more complicated in the ages 30–44 years (Fig. 7). A five-step in men and a two-step scheme in women denote the gender-specific element of this transition. At the same time, its pace is slower than for the younger ages. In males, while infant mortality, on average, changed between 1961 and 2019 with an AAPC of − 3.9%, the 1–14 age group with − 3.6% and the 15–29 age group with − 1.2%, in the ages 30–44 years it changed with an AAPC of -0.9% (the relevant values for females are − 4.0%, − 3.6%, − 2.2%, − 1.7% respectively; see Table 3 in the "Appendix").

This trend is also present in the age groups of 45–64 (AAPC = − 0.8%), 65–84 and 85–99 years (− 0.6% and − 0.2% respectively). For females, the applicable rates are − 1.3%, − 1.1% and − 0.0%. Thus, the overall transition tends to have a faster pace in females, and during people’s ageing process the annual mortality changes decelerate. However, as mortality gradually increases with age, these smaller annual percentage changes are accompanied by higher differences in the absolute change in the probabilities of death than in the younger ages.

The Annual Percentage Change (APC) differs between the different transition segments. In males of 30–44 years, the death probabilities initially decreased fast in 1961–1979 (APC = − 1.45%). This trend was reversed between 1986 and 1979 (APC = 0.84%). Several aggravating factors, including traffic accidents, decreased survival in these ages, burdened the population’s health and survival. APC reaches a maximum of -5.05% after the onset of the economic crisis, but any developments halt after 2014. In females, the transition scheme is more straightforward. Probabilities decline significantly until 1974 (APC = − 3.1), and their rate of decrease decelerates afterwards (− 1.31%).

In 45–64 years, the rate of mortality change (APC) in any segment is lower than in most previous ages. In males, a three-step scheme of mortality decline emerges, and APC exceeds 1% only for five years (1985–1990); therefore, the pace of transition is slow. This pace is faster in females, which, besides having lower mortality than males follow a different timetable of transition, seen in Fig. 7.

In the ages of 65–84 years, corresponding to the first group of older persons i.e., to the pensioners, the mortality transition is also slow, and only after 1998–1999 does it accelerate in both genders. In the very old ages of 85–99 years some fluctuations occur as expected mainly because of data quality problems discussed in the data and methods section of this paper. In males a minor decreasing trend is observed after 2011, while in females mortality increases after 2006 but with very low annual rhythms.

It seems then that the following major factors characterise the asymmetric mortality transition of Greece after 1961:

- The fast decrease of mortality rates in the younger ages.
- The slower annual pace of decrease of mortality in the older ages.
- The two genders have significant differences in their transitional course.

As a result, mortality rates of people less than 30 years old tend to become very low, while by the beginning of the economic crisis, a halt in the developments is observed in infants.

Consequently, the asymmetrical pace and timetable transition among the different age groups and the two genders results in any longevity trends in the most recent years being governed almost exclusively by changes in mortality in the older ages. The overall mortality transition in Greece (and consequently the phenomenon of rotation of mortality; see Christensen et al., 2009; Li et al., 2013) is connected primarily with the advances in the younger ages, and secondarily with the acceleration of mortality decrease in the older ages, of which the annual pace of reduction for the time being remains low. However, besides the annual pace of transition, as seen clearly in Fig. 7, these mortality shifts in absolute numbers are critical, and thus they govern solely the longevity changes seen in Fig. 3 and discussed previously.

### The death probability density distribution, the Gini index and the interquartile range

**Fig. 8:**
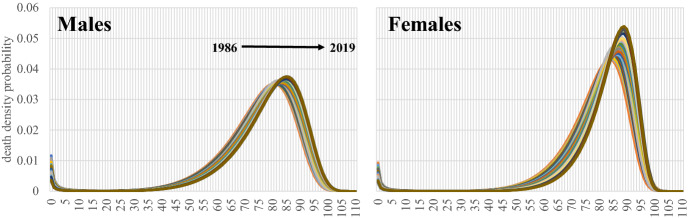
The death density probability distribution. Greece 1986–2019

The old-age heap of the death probability density distribution (g_x_, i.e., of the death curve) “moves” towards older ages in both genders over time. Simultaneously, the old age heap becomes narrower and higher (Fig. 8); thus, the survival curves become more rectangular.

**Fig. 9:**
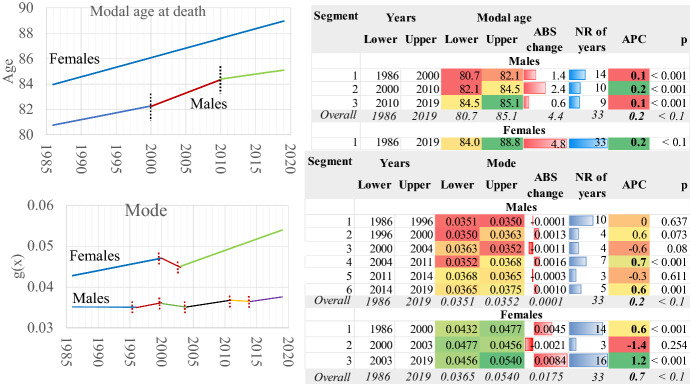
Modal age at death and Mode of the death density probability distribution. Greece 1986–2019. AAPC values are stored in row “Overall” and column “APC”

**Fig. 10:**
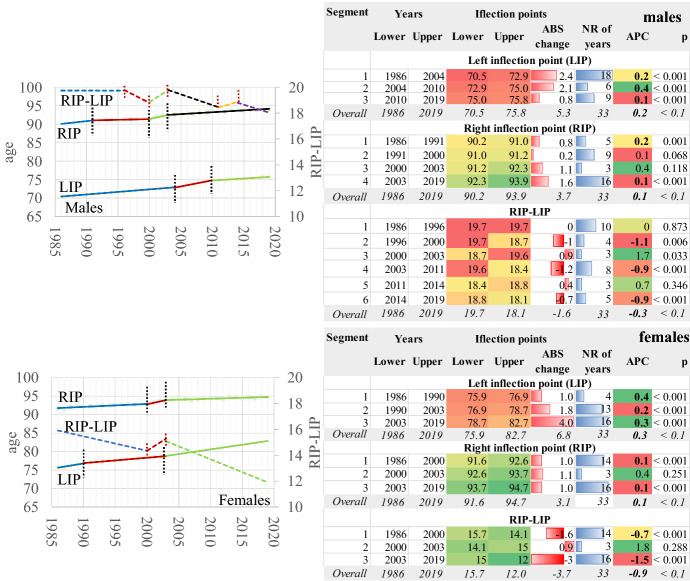
Left and right inflexion points and width of the death distribution in the old age death heap. Greece 1986–2019. AAPC values are stored in row “Overall” and column “APC”

This situation is described in Figs. 9 and 10. The modal age at death (Fig. 9), i.e., the age at which most deaths occur, changes linearly in females with an Annual Percentage Change (APC) of 0.2% or 4.8 years between 1986 and 2019. The change in males is lower, where the timetable of the developments is differentiated, and in fact, the most crucial effect is found in the 2000–2010 period. Besides these almost parallel developments, the mortality transition delays in males compared to females, and the modal age at death in 2019 is 3.7 years shorter. These estimations could serve perfectly as measurements of the average longevity change in the country, as they describe the developments in the old age heap in an environment of low infant and child to middle-age mortality.

The height of death curves is another rapidly changing characteristic, denoted in Fig. 9 as Mode of g(x). In women, the death curves become “higher” over time, increasing linearly. A transient but not statistically significant disturbance in the period 2000–2003 is replaced by a rapid increase later. Overall, the annual pace of increase (AAPC) is 0.7% between 1986 and 2019. For males, this increase is more moderate (AAPC = 0.2%), and the transition timetable is more complicated, another gender-specific characteristic of mortality transition in Greece. For females, the mortality transition has moved faster than males, and the developments in both modal age at death and mode are more rapid than in males.

The other gender-specific component is the width of the death curves at the old age heap (Fig. 10). The dynamic estimation of the left and right inflexion points and their age difference indicates significant limiting of the width of the death distribution in both genders. First of all, the left inflexion point is at a higher age in females and, overall, it changes faster than males towards older ages (AAPC = 0.2 in males and 0.3 in females). As a result, the left inflexion point became 9.1% higher in females in the year 2019. The transition took place in different steps in the two genders, contributing to the differentiation of mortality transition patterns between them.

The pace of change is slower for the right inflexion point (AAPC 0.1 for both genders), follow a different timetable of transition in males and females. The difference between the two genders is only 1% in 2019. Therefore, during the mortality transition in Greece, the left inflexion point moves faster than the right inflexion point. Characteristically enough, between 1986 and 2019, the left inflexion point changed 7.5% in males and about 10% in females, where the relevant values for the right inflexion point were 4.1% and 3.4%, respectively. This is an anticipated situation as it refers to the very old ages of the human life span, in which survivorship is limited, and the probabilities of death are very high. It should not be forgotten that the left inflexion point corresponds to the minimum speed of death distribution when the acceleration is 0. Afterwards, the acceleration increases, and the speed of death distribution gradually tends to be 0 when they are very high; therefore, the vitality of the organisms deteriorate even further compared with the right inflexion point, and any positive developments are very unlikely to occur.

The timetable of this transition (Fig. 10) is more complicated in males than in females. During the three-step procedure in females, the RIP-LIP age length decreases on average (AAPC) − 0.9% yearly, while the only period of disturbance is 2000–2003, when it increases slightly, but this is not statistically significant. In males, the temporal trends are more complicated, and overall, the length of the old age heap decreases only by 1.4 years between 1986 and 2019 (AAPC = − 0.3), compared with 3.7 years for females in this period. In fact, this variable changes for males in three periods: 1996–1987 (APC = − 1.1%), 2003–2011 (APC = − 0.9%) and 2014–2019 (APC = − 0.9%). The changes in other periods are not statistically significant.

Therefore, as the mortality transition progresses, the length of the old age heap becomes narrower. This transition is more advanced in females, and the LIP-RIP difference becomes much smaller than in males over time. As a result, the survival curves, which are the direct product of death distribution in a life table, tend to become more rectangular over time. This trend is more intense in females; however, one could easily hypothesise that males will follow in the future if this trend of mortality reduction prevails.

**Fig. 11:**
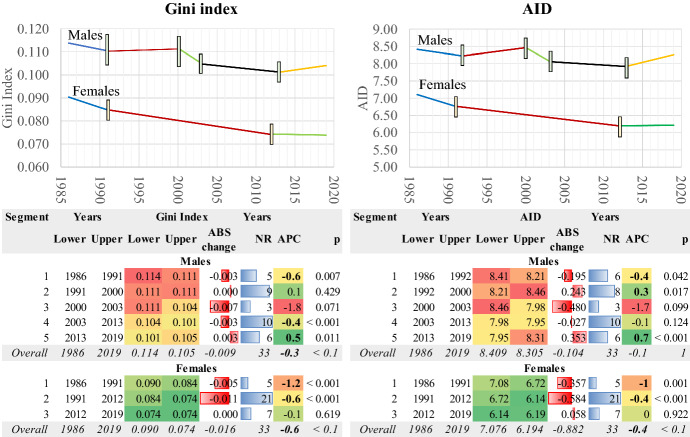
The Gini coefficient (index) and the average inter-individual difference (AID). Greece 1986–2019. AAPC values are stored in row “Overall” and column “APC”

These findings are confirmed by using the Gini Coefficient (index) and the average inter-individual difference (AID; Fig. 11). Note that the higher the Gini index, the higher the mortality inequality among the population members (see, for example, Peltzman, 2009; Skaftun et al., 2018) and vice versa. The Gini index decreased from 0.09 to 0.074 in 33 years or about 18%, with an AAPC-0.6% per year in females. A similar decreasing trend is found for the average inter-individual differences (AID). Both variables follow a stepwise decreasing scheme of transition. Therefore, the dispersion in deaths decreased over time, and the inequality in the age of death became smaller. After 2012, just after the onset of the economic crisis, any developments halt.

The changes initially are more complicated for males, but after 1991 until 2003, none of the linear regression lines was significant. On the contrary, the Gini Coefficient changed considerably between 1986 and 1991 (APC = − 0.6%) and 2003–2013 (APC = − 0.4%). After 2013 the Gini coefficient increases; therefore, the inequality becomes larger. In both genders, these developments occur against a background of economic crisis, which might be a reason for their formulation.

**Fig. 12:**
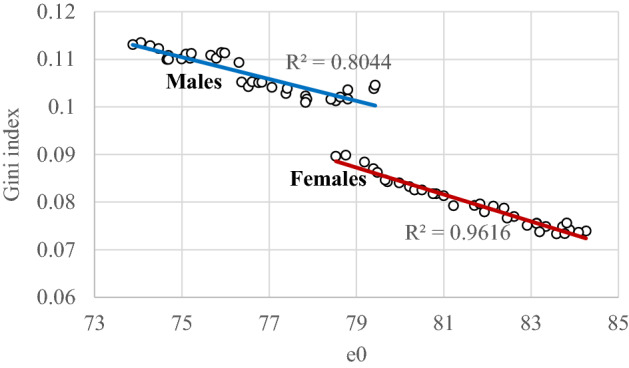
The life expectancy at birth (e0) and Gini coefficient (index). Greece 1986–2019

The relation between average longevity and the Gini coefficient is seen in Fig. 12. It seems that as the mean duration of life (e0) increases, mortality inequality decreases. This is a typical situation for human populations; Colchero et al. (2016) indicate that as lifespan increases, the relative variation in lifespan decreases (for a discussion on the basis of worldwide data see Smits & Monden, 2009). This trend is more prominent in the country’s female population, even if a halt in the decline of the Gini coefficient is observed in the last years, as already mentioned above. In males, the reversal of the downward trend in recent years and their general differences compared to women, results in a less clear linear course over time. It must be noted that the temporal trends of the Gini coefficient in Greece, its decomposition by age and cause of death and several other aspects will be discussed in a forthcoming paper.

**Fig. 13:**
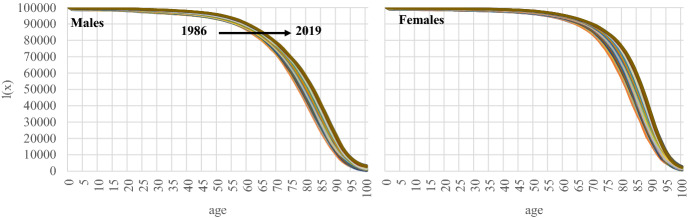
The survival curves (lx). Greece 1986–2019

The rectangularization trend of the survival curves, described in the previous section, is seen in Fig. 13 in the "Appendix". As happened with the death curves, these “tend” to move towards older ages and become steeper over time. This phenomenon is more pronounced in females than males, as the transition has moved well ahead in the former.

**Fig. 14:**
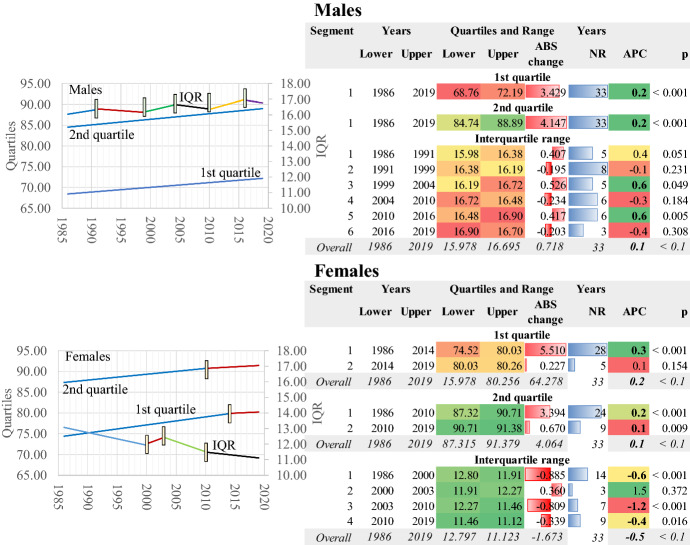
The quartiles of the survival curve and the interquartile range. Greece 1986–2019. AAPC values are stored in row “Overall” and column “APC.”

The characteristics of this process can be described by the quartiles and the interquartile range (Fig. 14 in the "Appendix"). However, as the conclusions emerging from this analysis are similar to those discussed in the previous section of this paper, Fig. 14 is only cited here to verify the previous findings.

### The major causes of death

**Fig. 15:**
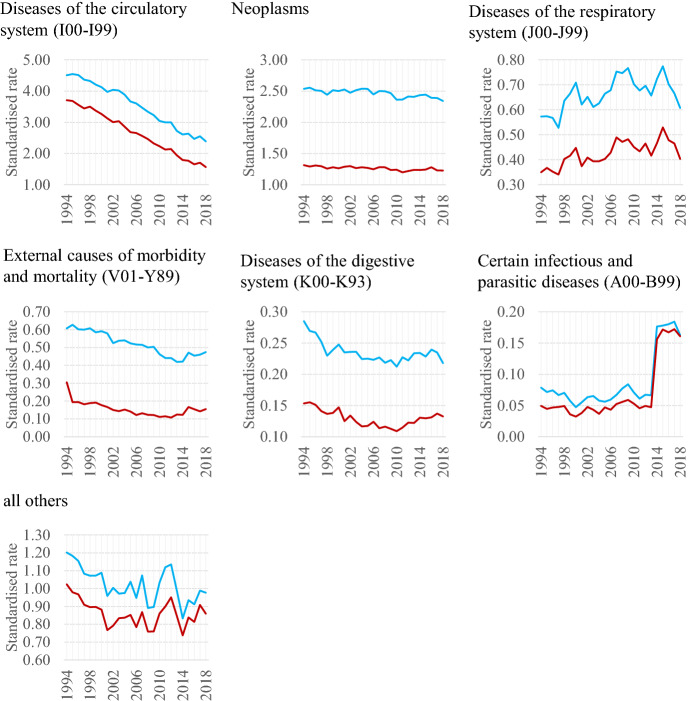
The major causes of death. Directly standardised rates. Greece 1994–2019

The mortality transition described in the previous sections is the result of the differential effect of the burden of different diseases (Table 1). The standardised rates of the major causes of death are seen in Fig. 15 in the "Appendix". These causes of death are ranked by their levels; thus, their relative impact on mortality can be assessed).

**Table 1:**
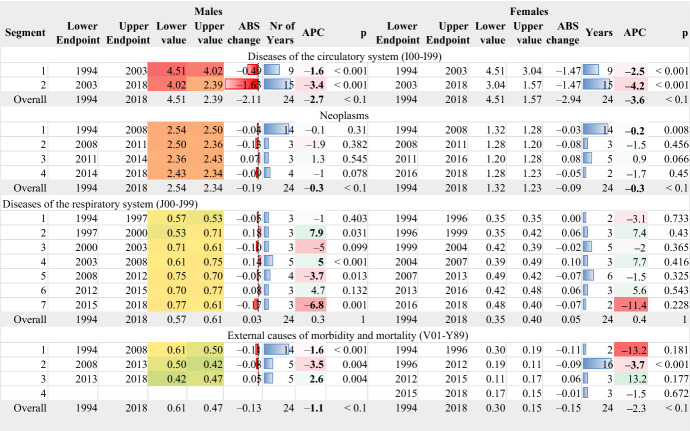
Standardised rates in the four most crucial aggravating factors of longevity

AAPC values are stored in row “Overall” and column “APC”

A previous paper presented the effects of tobacco-related diseases on mortality and longevity following a decomposition procedure (Zafeiris, 2020a); thus, any findings will not be discussed here. Also, the age-standardised rates have been presented before, but no particular focus was made concerning their temporal trends (see Kotzamanis et al., under publication).

The most important of these causes are the diseases of the circulatory system. Overall, mortality rates decrease faster in females, but the two genders share a similar transition timetable (Table 1), with this transition accelerating after 2003. The AAPC for males is − 2.7% and for females − 3.6%. Lifestyle agents (see WHO, 2007) are responsible for the high prevalence of these diseases in Greece, including smoking, air pollution, obesity, lack of physical activity and others (see also Zafeiris, 2020a). In the year 2011, increased and effective medical treatment of this group of diseases in Greece led to a reduction in their lethal effects to much lower levels than in other European countries, though they remained at higher levels compared with the EU15 countries, i.e., the ones belonging to the European Union before May 1st 2004 (see WHO, nd).

The second group of diseases affecting mortality and longevity in Greece are neoplasms (Fig. 15, Table 1). The standardised rates of these diseases remain essentially unchanged over time in both genders, remaining in 2011 very close to those of EU15 and the other European countries (WHO, nd).

The third most important factor is respiratory system diseases. These fluctuate a lot over time, but with a general trend of increase in both genders, which is more pronounced in males. The APC values in this transition are never significant in females. In the last years of the study, corresponding to the economic crisis, a sharp decrease in the standardised rates indicates (statistically significant only in males) the improvements in reducing the risk factors (i.e., smoking, air pollution and others) in the onset of these diseases.

Many of these diseases described above can be characterised as amenable or preventable. Diseases that are amenable could have been avoided if the quality of the health care system was at its optimal state. Preventable diseases could have been prevented if people’s behaviour and lifestyle, socio-economic status and environmental agents changed through public interventions (EUROSTAT, 2018). Therefore, these findings stress the need for further enhancement of public policies for improving the health of the population in the country.

The same can happen with the fourth major cause of death, i.e., the external causes of mortality and morbidity. For most of the years studied, these causes of death decreased in the two genders. This group of diseases includes traffic accidents, suicides and other reasons. The impact of traffic accidents has been discussed previously, but it is worth noting that suicides increased during the economic crisis (see, for example, Rachiotis et al., 2015). This fact may explain the elevation of mortality rates in males in the period 2013–2018.

The following two major causes of death (digestive system diseases and certain infectious and parasitic diseases; Fig. 15 and Table 2) are less important. The diseases of the digestive system decrease over time, while a different timetable of transition is observed between the two genders. The rapid increase in infectious and parasitic diseases after 2012 corresponds to seasonal influenza outbreaks (see Lytras et al., 2019). All the other causes of death have been summed in the category “all others”, and they tend to decrease over time, although not without significant temporal fluctuations.

**Table 2:**
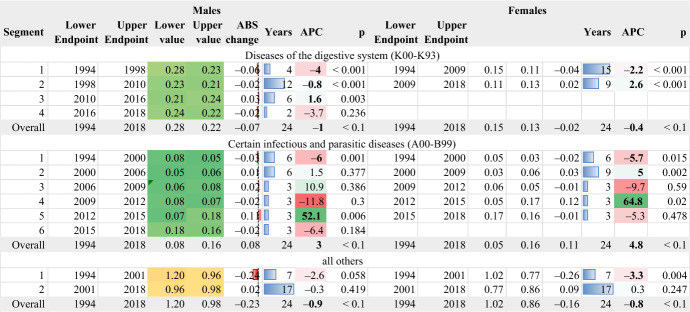
Standardised rates in the rest major causes of death

AAPC values are stored in row “Overall” and column “APC”

## Discussion and conclusions

Life in Greece becomes longer and healthier over time. Thus, a stepwise increase in life expectancy at birth is observed since 1961. This trend continued in males after the emergence of the economic crisis. In females, whose average longevity was already high, any developments halt, but this can hardly be attributed to the crisis. Arriaga’s decomposition procedure revealed that as the mortality transition in Greece continues, the mortality developments in the older ages play an increasingly important role in regulating longevity trends. In contrast, the role of younger ages becomes of lesser importance.

This phenomenon, known as the “rotation of mortality”, is connected in Greece to the initial acceleration of mortality transition in people younger than 30 years. Mortality transition in the other ages exhibits lower rhythms of change over time, but in absolute values is pronounced because it comes from increased probabilities of death. Therefore, it exclusively governs the longevity developments in the more recent era.

While similar observations can be made for life expectancy at different ages, it is worth noting that after the advent of the economic crisis, the pace of improvement for ages 15 and 30 years tended to accelerate due to mortality developments in the accident hump. In the rest of the ages, mixed trends prevail in the pace of transition. Overall, longevity shifts in various ages depend on age and gender.

This is directly related to the temporal developments in the probabilities of death in the various age classes studied. An asymmetrical scheme emerges for this transition. As observed in life expectancy in various ages, this asymmetry has an age and a gender-specific component, including the differentiated temporal trends between the older (slower pace of decrease but higher changes in absolute values) and younger ages (faster pace of decrease) and the specialisation of these trends in the two genders. While these trends have been discussed previously, some effects of the economic crisis on mortality have been identified. Infant mortality tends to increase after the onset of the economic crisis, noting at the same time its very low levels.

Also, mortality in ages 15–29 years is much higher in males, but after 2009 the economic crisis limited financial capabilities and risky behaviours. As a result, the mortality rates in this age class diminished rapidly.

The analysis of the death curve revealed the increase of modal age at death and the mode of distribution over time. This process is faster in the country’s female population, in which the mortality transition is more advanced. Thus, as the modal age at death increases, the death curve becomes higher. At the same time, the left and right inflexion points move towards older ages but at a different pace. The left inflexion point moves faster than the right inflexion point. In females, both inflexion points are located in older ages. As a result, the width of the old age heap becomes narrower over time, while the old age death heap moves towards older ages.

Consequently, deaths tend to concentrate more around the modal age at death, a phenomenon known in the literature as “compression of mortality” (see for example, Kannisto, 2001; Thatcher et al., 2010). However, at the same time, the mortality patterns in the old age heap shift towards older ages, while variability in age of deaths reduces, a probable and transient departure from the “shifting mortality hypothesis” as the compression of mortality has not stopped yet (see Canudas-Romo, 2008; Bergeron-Boucher et al., 2015). This process is much more advanced in the female population of Greece.

In any case, the survival curves become more rectangular (see also the interquartile range) over time as the variability in the ages of death decreases while the old age death heap is moving towards older ages (see also Wilmoth, 1997; Wilmoth & Horiuchi, 1999). Accordingly, the Gini Index (Coefficient) and the inter-individual difference (AID) decrease in both genders. Once again, this trend is more pronounced in females. However, another effect of the economic crisis on mortality is located here. In females, in the last years of the study (i.e., after the onset of the economic crisis), the decreasing trend of the variability in the age of deaths stops. In males, this variability increases. It seems then that the economic crisis affected the two genders differentially, either by disturbing the death age distribution or by keeping it unchanged.

The prevalence of several fatal diseases regulates the trends mentioned above. Of them, the diseases of the circulatory system are the most important. However, their rates decrease steadily over time in both genders. The pace of decrease is higher in females. The second most crucial regulating factor is the neoplasms, the effects of which do not change much of the time.

In contrast, diseases of the respiratory system increased over time until the advent of the economic crisis. This trend is more pronounced in males, on whom the economic crisis had positive effects. These diseases are amenable or preventable, a fact which denotes the necessity of further enhancement of public policies for improving health status in Greece. The fourth most important cause of death is external causes of morbidity and mortality. It is worth noting that the previously observed declining trend diminished some years after the emergence of the economic crisis. Undoubtedly the increase in suicides played a significant role in that.

## Appendix

See Table 3 and Figs. 13, 14, 15.

**Table 3:** Average annual percent change (AAPC) of the probabilities of death per age class and gender between 1961 and 2019

Age class	Males		Females	
	AAPC	*p*	AAPC	*p*
q0	− 3.9	< 0.1	− 4.0	< 0.1
1–14	− 3.6	< 0.1	− 3.6	< 0.1
15–29	− 1.2	< 0.1	− 2.2	< 0.1
30–44	− 0.9	< 0.1	− 1.7	< 0.1
45–64	− 0.8	< 0.1	− 1.3	< 0.1
65–84	− 0.6	< 0.1	− 1.1	< 0.1
85–99*	− 0.2	< 0.1	− 0.0	< 0.1

*The estimations for this age class refer to the 1986–2019 period
